# Maladaptive striatal plasticity and abnormal reward‐learning in cervical dystonia

**DOI:** 10.1111/ejn.14414

**Published:** 2019-05-14

**Authors:** Tom Gilbertson, Mark Humphries, J. Douglas Steele

**Affiliations:** ^1^ Department of Neurology Ninewells Hospital & Medical School Dundee UK; ^2^ Division of Imaging Science and Technology Medical School University of Dundee Dundee UK; ^3^ Division of Neuroscience & Experimental Psychology University of Manchester Manchester UK; ^4^ School of Psychology University of Nottingham Nottingham UK

**Keywords:** basal ganglia, cervical dystonia, cortico‐striatal plasticity, reinforcement learning, reward prediction error

## Abstract

In monogenetic generalized forms of dystonia, in vitro neurophysiological recordings have demonstrated direct evidence for abnormal plasticity at the level of the cortico‐striatal synapse. It is unclear whether similar abnormalities contribute to the pathophysiology of cervical dystonia, the most common type of focal dystonia. We investigated whether abnormal cortico‐striatal synaptic plasticity contributes to abnormal reward‐learning behavior in patients with focal dystonia. Forty patients and 40 controls performed a reward gain and loss avoidance reversal learning task. Participant's behavior was fitted to a computational model of the basal ganglia incorporating detailed cortico‐striatal synaptic learning rules. Model comparisons were performed to assess the ability of four hypothesized receptor specific abnormalities of cortico‐striatal long‐term potentiation (LTP) and long‐term depression (LTD): increased or decreased D1:LTP/LTD and increased or decreased D2: LTP/LTD to explain abnormal behavior in patients. Patients were selectively impaired in the post‐reversal phase of the reward task. Individual learning rates in the reward reversal task correlated with the severity of the patient's motor symptoms. A model of the striatum with decreased D2:LTP/ LTD best explained the patient's behavior, suggesting excessive D2 cortico‐striatal synaptic depotentiation could underpin biased reward‐learning in patients with cervical dystonia. Reversal learning impairment in cervical dystonia may be a behavioral correlate of D2‐specific abnormalities in cortico‐striatal synaptic plasticity. Reinforcement learning tasks with computational modeling could allow the identification of molecular targets for novel treatments based on their ability to restore normal reward‐learning behavior in these patients.

## Graphical abstract

Here we demonstrate that reward‐learning in patients with cervical dystonia is abnormal and that this can be directly related to impaired plasticity at the cortico‐striatal synapse. These results support the idea that a common link between focal and generalized forms of dystonia (where animal models have provided direct evidence aberrant of striatal plasticity) is the presence of abnormal cortico‐striatal plasticity. In the future, treatments which target this may be used clinically to alleviate symptoms of this common movement disorder.

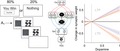

**Abbreviations**
CD: cervical dystonia
CTRL: control
LTD: long‐term synaptic depression
LTP: long‐term synaptic potentiation
MNI: Montreal Neurological Institute
RPE: reward prediction error

## 1 INTRODUCTION

Cervical dystonia is a form of primary focal dystonia characterized by involuntary muscle contractions and posturing of the neck region that leads to significant disability and pain. The degraded action selection which underpins these involuntary movements have long been thought to be the consequence of dysfunction within the basal ganglia, based on clinical observation (Berardelli et al., 1998; Bhatia & Marsden, 1994), neuroimaging (Colosimo et al., 2005; Draganski et al., 2009; Naumann et al., 1998; Zoons, Booij, Nederveen, Dijk, & Tijssen, 2011), and its secondary association with “extra‐pyramidal” disorders (Burke et al., 1982; Louis, Lee, Quinn, & Marder, 1999; Rivest, Quinn, & Marsden, 1990). The recent identification of families with novel mutations in genes which encode proteins richly expressed within the striatum implicated in dopaminergic signal transduction, e.g., GNAL (Kumar et al., 2014), and ANO3 (Charlesworth et al., 2012) has reignited interest in “old ideas” about dopaminergic dysfunction being a common pathophysiological hallmark (Goodchild, Grundmann, & Pisani, 2013). For patients with cervical dystonia, detailed molecular and neurophysiological understanding of the nature of this derangement in signaling is crucial for the development of new treatments.

By its opposing effects on cortico‐striatal plasticity at D1 and D2 receptors (Surmeier, Ding, Day, Wang, & Shen, 2007) dopamine determines which actions are selected for a specific context, by encoding action values in cortico‐striatal synaptic strengths (Samejima, Ueda, Doya, & Kimura, 2005). If dopamine signaling is abnormal in patients with cervical dystonia, one potential cause for the breakdown in action selection is the downstream effect of abnormal signaling on synaptic connectivity at the cortico‐striatal synaptic interface (Peterson, Sejnowski, & Poizner, 2010; Quartarone & Pisani, 2011). Abnormalities in striatal plasticity are thought to be a pathogenic mechanism common to all forms of dystonia (Peterson et al., 2010). In animal models of rare, childhood onset generalized dystonia (DYT1, DYT6), highly specific cortico‐striatal plasticity abnormalities have been observed in vitro *(*Maltese et al., 2017 *;* Martella et al., 2009 *;* Pisani et al., 2006; Zakirova et al., 2018). Whether defective cortico‐striatal plasticity contributes to the expression of the common late onset focal forms, including cervical dystonia, remains unknown.

We therefore developed an experimental paradigm and computational model that aimed to test two hypotheses: (a) cervical dystonia is associated with a measurable bias in reward‐learning (b) a specific abnormality in cortico‐striatal plasticity could explain this abnormal behavior.

## 2 MATERIALS AND METHODS

### 2.1 Participants

The study was approved by the local Ethics Committee (East of Scotland Research Ethics Service, reference number 2014NG03) and written informed consent obtained from all volunteers. A total of 80 subjects including 40 healthy controls and 40 patients with cervical dystonia were studied. All patients were recruited from movement disorder clinics from four regional neuroscience centers in Scotland (Aberdeen, Dundee, Edinburgh and Glasgow). The diagnosis of cervical dystonia was made by a Consultant Neurologist with a specialist interest in movement disorders. Patients were all receiving botulinum toxin injections and behavioral testing was performed to coincide with maximal treatment response (2–8 weeks). The principal exclusion criteria for the study were: secondary dystonia and drug induced dystonia and previous or ongoing mood, anxiety, or other psychiatric disorders. Patients taking anti‐cholinergic or other centrally acting pharmacological agents for treatment of their cervical dystonia were excluded. Age, IQ, and sex‐matched controls were university and NHS staff members with no previous history or active symptoms of neurological or psychiatric disease.

#### 2.1.1 Rating scales

Clinical rating of cervical dystonia severity was assessed using the Cervical Dystonia Impact Profile (CDIP‐58) (Cano et al., 2004). Ratings of mood, anxiety, and obsessive‐compulsive symptoms were derived using the HADS and Y‐BOCS assessments (Goodman et al., 1989; Zigmond & Snaith, 1983). The NART was used to estimate IQ (Nelson & Wilson, 1991).

#### 2.1.2 Behavioral task

**Figure 1:**
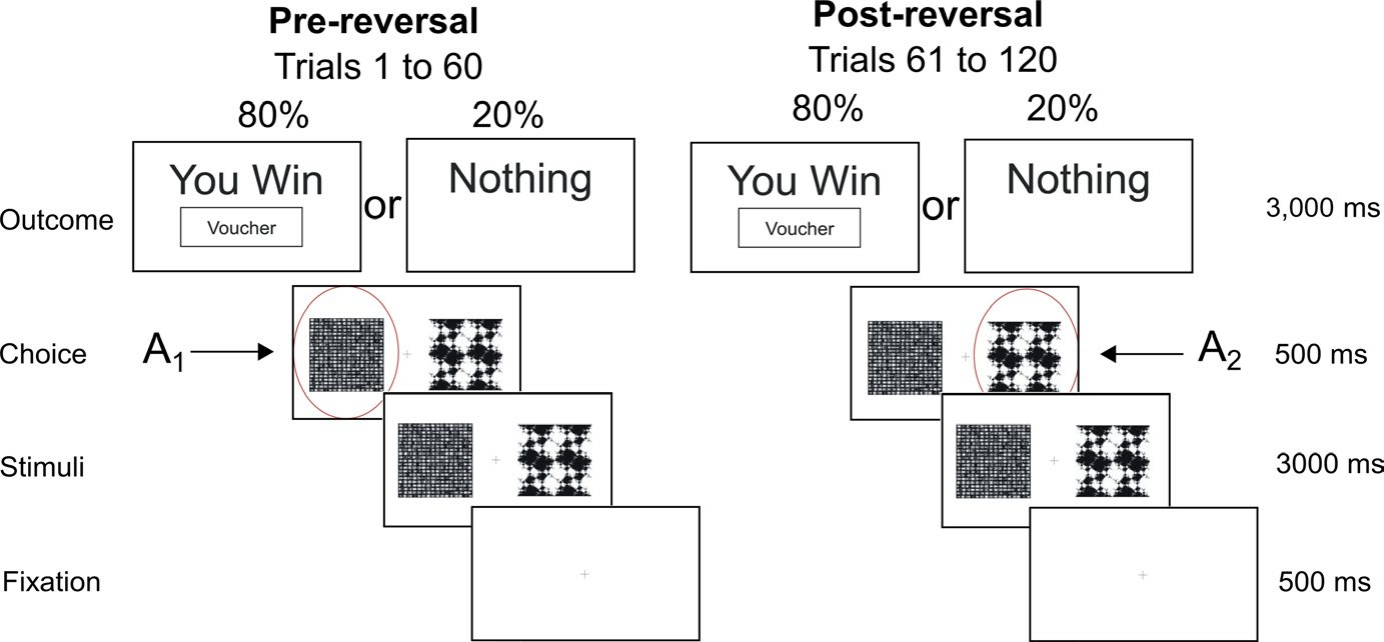
Probabilistic reversal learning task. Example of fractal images presented during a single reward trial. The probability of receiving a reward “voucher” reverses after 60 trials requiring the participant to suppress the previously learnt choice and learn to “reverse” their decision to choose the previously low value (pre‐reversal) fractal. In the pre‐reversal phase choice of A_1_ is associated with an 80% outcome of reward. Post‐reversal this reduces to 20% with the choice of A_2_ being associated with 80% [Colour figure can be viewed at http://wileyonlinelibrary.com]

Patients and controls performed a probabilistic reward (Figure 1) and loss (Figure S1) reversal learning task based on previous reinforcement learning studies (Gradin et al., 2011; Pessiglione, Seymour, Flandin, Dolan, & Frith, 2006). Subjects were asked to learn from trial and error in order to obtain “vouchers” and were informed at the beginning of the task that their voucher score would be converted into money on completion. This ranged from £20–30 depending on the number of vouchers obtained. During the task one set of two pairs of fractal images were presented on a computer screen. Subjects chose one of the two fractals with a button press with the aim of maximizing wins (positive reinforcement) and minimizing losses (negative reinforcement) by trial and error. Trials where responses were not obtained within 2.5 s were not included in the analyses. Feedback following image choice informed subjects as to whether they “won” or “lost” a voucher (in the reward and loss trials respectively) or “nothing” where no change in score occurred. Reward and loss trials were presented randomly with an 80:20 probability of a reward or loss being the outcome with a total of 120 trials per task being presented. After 60 trials, the contingencies were reversed, requiring subjects to extinguish their previously learned action–outcome relationship. The task was performed over three sessions with short between‐session breaks and the reversal occurring midway through the second session. A 5‐min training session was performed before formal testing began.

The motivation behind our choice of task was the recognized role of the basal ganglia and in particular the striatum in reversal learning (Bellebaum, Koch, Schwarz, & Daum, 2008; Cools, Clark, Owen, & Robbins, 2002). Reversal learning involves the initial acquisition and subsequent (post‐reversal) extinction of action–outcome contingencies. These processes depend upon both direct (D1) and indirect (D2) pathway medium spiny neurons (MSN's) (Cools et al., 2009; Cox et al., 2015; Nakanishi, Hikida, & Yawata, 2014). By using a task which engages both striatal populations, our aim was to elucidate specific isolated or combinations of abnormalities, in both D1 and D2 plasticity. The paradigm was programmed using MATLAB (MathWorks, Natick, MA) with PsychToolbox (Brainard, 1997).

### 2.2 Functional MRI data acquisition and image analysis

Functional MRI was acquired in twenty of the controls and nineteen of the patients performing the task (one patient was unable to tolerate scanning due to claustrophobia, but completed the behavioral experiment outside the scanner). For each participant, functional whole‐brain images were acquired using a 3T Siemans Magnetom TimTrio Syngo scanner using an echo planar imaging sequence with the following parameters: angle = 90°, field of view = 224 mm, matrix = 64 × 64, 37 slices, voxel size 3.5 × 3.5 × 3.5 mm. Images where visually inspected for artefacts and preprocessing was performed in SPM8 (http://www.fil.ion.ucl.ac.uk/spm). Images were realigned and co‐registered to the SPM8 Montreal Neurological Institute echo planar imaging template. The average, realigned co‐registered image for each subject was used as a template to normalize each realigned and co‐registered volume to the SPM8 echo planar imaging template image before smoothing. For first level analysis, an event‐related model‐based analysis was implemented with onsets at the outcome time (when the subject received feedback as to whether their choice was successful or not). The reward prediction error (RPE) signal generated from the individual subjects optimally fitted “Standard Reinforcement Learning Model” (see below) was used to parametrically modulate a truncated delta function convolved with the hemodynamic response function. Second‐level analysis was restricted to one‐group *t*‐tests to assess for significant activation patterns within groups and two‐group *t*‐tests for estimates of between‐group (patient vs. controls) differences in RPE activation patterns. Using a popular Monte‐Carlo method (Slotnick, Moo, Segal & Hart, 2003), significance was defined as *p *< 0.01 at a whole brain, Family‐Wise error corrected level, achieved by a simultaneous requirement for a *p* < 0.05 voxel threshold and a cluster extent >120 voxels.

### 2.3 Computational modeling

**Figure 2:**
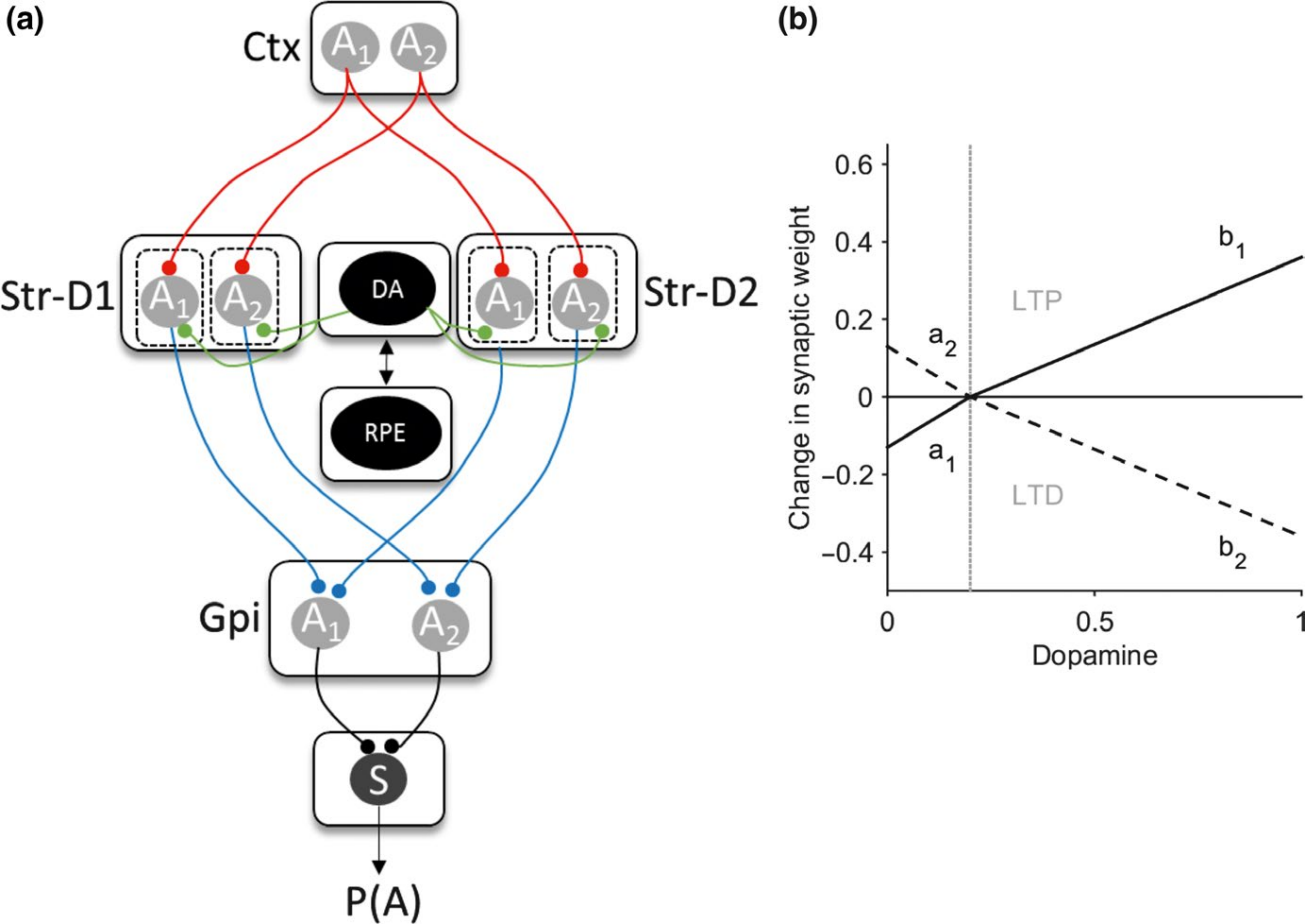
Basal ganglia plasticity model. (a) Schematic model architecture. Nuclei include the direct pathway striatal neurons (Str‐D1), indirect pathway (Str‐D2), and globus pallidus interna (GPi). Gray circles represent the available two actions (*A*_1_ and *A*_2_) represented in the cortex (Ctx) and subcortical nuclei. Red lines represent excitatory connections, blue inhibitory. Green represents the neuromodulatory influence of dopamine on the striatum. Functions critical to the model include the dopamine‐weight change function (DA), the reward prediction error signal (RPE), and the softmax (S) equation. The value of an action is assumed to be represented in the cortex (Ctx) for one of two actions (*A*_1_ and *A*_2_). The striatal output is determined by the product of the cortico‐striatal synaptic weight and the cortical input for the action. The NoGo activity of one action was subtracted from its “Go” D1” self (i.e., Gpi(A1) = StrD1(A1)−StrD2(A1). The pallidal activity for each action is then converted into a choice probability *p*(*A*) by entering this into the softmax function (S). (b) The expected change in synaptic weight as a function of dopamine released is represented by the dopamine‐weight change curve (DA). The four plasticity coefficients (*a*_1_, *b*_1_, *a*_2_, *b*_2_) in the model determine the gradients of this function, which in turn govern the magnitude of LTP and LTD at the cortico‐striatal synapse. The solid line represents the D1 dopamine‐weight changes, the dashed line the D2 equivalent. The gray vertical line represents the point of inflection at which dopamine is considered above or below baseline levels, corresponding to phasic “bursts” or “dips” in the reward prediction error signal [Colour figure can be viewed at http://wileyonlinelibrary.com]

Two models were fitted to the observed behavior. In the first instance, a standard reinforcement learning model was chosen (Sutton & Barto, 1998) which robustly captures phasic dopamine neuron firing in the form of a RPE. The dynamics of the RPE signal in this model is determined by estimating two parameters, the learning rate (*α*) and reward sensitivity (*β*). This type of model has been used previously to explore abnormal presynaptic phasic dopamine signaling in pathological states such as Parkinson's disease (Peterson et al., 2009). In contrast, presynaptic signaling is thought to be preserved in cervical dystonia with postsynaptic D2 receptor expression impaired (Naumann et al., 1998). Our aim therefore was to test the hypothesis that postsynaptic cortico‐striatal plasticity was abnormal. To that end, we developed a detailed basal ganglia model (Figure 2a) which combined phasic dopamine signaling with cortico‐striatal synaptic plasticity dynamics (Gurney, Humphries, & Redgrave, 2015). This model required estimation of six parameters: learning rate (*α*) and reward sensitivity (*β*), plus four “plasticity coefficients” (*a*_1_, *b*_1_, *a*_2_, *b*_2_). Each coefficient determined the relative influence of phasic dopamine release on the cortico‐striatal synaptic weight; with *a*_1_, *b*_1_ scaling D1 long‐term depression (LTD) and D1‐LTP, and *a*_2_, *b*_2_, scaling the D2 LTP and D2‐LTD synaptic changes, respectively (Figure 2a).

#### 2.3.1 Standard reinforcement learning model

This trial‐by‐trial reforcement learning model robustly captures the dynamics of phasic dopamine neuron firing in the form of a RPE represented by (*R*(*t*)−Q(*A*,*t*−1)) in the equation;

(1) Q(A,t)=Q(A,t−1)+α(R(t)−Q(A,t−1))

where the *Q*‐value of action A in on trial *t* is the *Q*‐value of the action on the previous trial (*t *− 1) updated by the RPE. *R*_t_ the outcome (reward[1] or nothing[0]) and *α* is the learning rate. The choice of action in the trial‐by‐trial reinforcement learning model is determined by the sigmoid or softmax function, where the probability *p* of choosing action *A*_1_ over action *A*_2_ is defined as:

(2) $$P\left(A_{1},t\right)=\frac{e^{\beta Q(A_{1},t-1)}}{e^{\beta Q(A_{1},t-1)}+e^{\beta Q(A_{2},t-1)}}$$

with *β* the reward sensitivity or inverse temperature parameter. Parameters where estimated using a random effects, expectation maximization procedure implemented in Matlab code provided by QJM Huys (https://www.quentinhuys.com/tcpw/code/emfit/).

To test for differences in the modeled RPE signal, individual subject estimates of the learning rate (*α*) and reward sensitivity (*β*) for patients and controls were generated. These were then used to test the null hypothesis of no significant difference in the model parameter estimates between groups. Comparative statistics were performed on normalized parameter estimates (Huys, Pizzagalli, Bogdan, & Dayan, 2013) where the normalized learning rate is a normally distributed random variable with mean −1 and related to the learning rate, *α*, by the following:

$$\alpha =\frac{1}{1+e^{-x}}$$

#### 2.3.2 Basal Ganglia model

A model was developed which incorporated a suitable level of detail to allow inference of striatal synaptic plasticity abnormalities, while being simple enough that it could be fitted to behavioral data. This “basal ganglia” model was designed to extend any general conclusions, inferred from a trial‐by‐trial reinforcement learning model, into more detailed mechanistic abnormalities that could be related to dopamine's role in biasing action selection in the basal ganglia.

At the core of the basal ganglia model (Figure 2a) was a hybrid approach, incorporating standard reinforcement learning rules (to produce the RPE from Equations  and with cortico‐striatal synaptic plasticity parameters, derived from the model of Gurney et al. (2015). Aiming for a simple parsimonious model, two striatal “populations” were assumed *S*_D1_ and *S*_D2;_ the D1 receptor expressing direct and D2 receptor expressing indirect pathways respectively. The striatal activity of each population *n*, on trial *t* for action *A* was:

(3) Sn(A,t)=Wn(A,t−1)·c,

where *W* is the cortico‐striatal synaptic weight and *c* is a constant input of 1. Here we use a model with two actions (*A*_1_, *A*_2_) corresponding to the choice between the two options.

Competition between the two striatal pathways for control of basal ganglia output was of most interest. Thus, the pallidal output for action *A*_1_ was defined as:

(4) $$\begin{array}{cc} & \text{GPi}(A_{1},t)\\ & \;=\left(S_{D1}\left(A_{1},t\right)-S_{D2}\left(A_{1},t\right)\right)H\left(S_{D1}\left(A_{1},t\right)-S_{D2}\left(A_{1},t\right)\right) \end{array}$$

where *H()* is the Heaviside step function: *H*(*x*)* = *0 if *x *≤ 0, and *H*(*x*)* =* 1 otherwise; and similarly for action *A*_2_. The pallidal output was then proportional to the amount of disinhibition of the basal ganglia's targets. In turn the probability of choosing action *A*_1_ was determined by the softmax equation with the basal ganglia's output substituted for the value term:

(5) $$P\left(A_{1},t\right)=\frac{e^{\left(\mathrm{GPi}\left(A_{1},t\right)/\beta \right)}}{e^{\left(\mathrm{GPi}\left(A_{1},t\right)/\beta \right)}+e^{\left(\mathrm{GPi}\left(A_{2},t\right)/\beta \right)}}$$

The cortico‐striatal synaptic weights are assumed to take on only positive values and are modified at the synapse corresponding to the chosen action *A*

(6) $$\begin{array}{cc} & W_{n}\left(A,t\right)\\ & =\left\{\begin{array}{cc} W_{n}\left(A,t-1\right)+\Delta W_{n}\left(A,t-1\right), & \text{if}\;W_{n}\left(A,t-1\right)+\Delta W_{n}\left(A,t-1\right)>0\\ 0, & \text{otherwise} \end{array}\right. \end{array}$$

The change in synaptic weight is assumed to obey a Hebbian two‐factor rule as the product of the striatal postsynaptic activity (*S*) and the neuromodulatory influence of dopamine (∆*d*_*n*_(*t*)) assuming constant (presynaptic) cortical input:

(7) $$\Delta W_{n}\left(A,t\right)=\Delta d_{n}\left(t\right)\cdot S_{n}\left(A,t\right)$$

The extent of dopamine related changes in synaptic strength are governed by separate *D*1 and *D*2 functions following the detailed model of dopamine‐modulated plasticity in Gurney et al. (their fig. 13), and capture the dependence of the sign and magnitude of synaptic change on both the concentration of dopamine (DA) and the receptor‐type of the postsynaptic striatal neuron (D1 or D2).

For the general case of a *D*_*n*_ dopamine receptor subtype, the magnitude ∆*d*_*n*_ of dopamine's effect on synaptic plasticity is,

(8) $$\Delta d_{n}\left(t\right)=\left\{\begin{array}{cc} a_{n}\left(\text{DA}\left(t\right)-\theta \right), & \;\text{if}\text{DA}(t)>\theta \\ b_{n}\left(\text{DA}(t)-\theta \right), & \;\text{otherwise} \end{array}\right.$$

where (*a*_*n*_, *b*_*n*_) are coefficients determining the dependence of synaptic plasticity on the current trial's level of dopamine DA(t), and the constant θ determines the baseline level of dopamine. Following the predictions of Gurney et al. (2015), for D1 neurons we expect here (*a*_1_, *b*_1_) >0, so that dopamine levels below baseline cause a decrease in cortico‐striatal weight (LTD), and dopamine levels above baseline cause an increase in weight (long‐term potentiation; LTP); for D2 neurons we expect here (*a*_2_, *b*_2_) < 0, thus giving LTP below baseline, and LTD above it. However, in fitting this model to the behavioral data, we did not restrict the sign of the coefficients.

Following standard RPE accounts of dopamine signaling, values of DA >*θ* are phasic increases, a positive prediction error, whereas DA <*θ* represent phasic dips in dopamine, the negative prediction error signal. Thus, the RPE(*t*) signal of Equation  was mapped to DA(*t*) in Equation  as:

(9) $$\text{DA}\left(t\right)={\text{DA}}_{\min}+\frac{\left(\text{RPE}\left(t\right)-{\text{RPE}}_{\min}\right){\text{DA}}_{\mathrm{range}}}{{\text{RPE}}_{\mathrm{range}}}$$

To map the RPE(*t*) signal in the range [−1,1] to the DA value in [0,1] around the baseline of *θ*, we use: if RPE(*t*) <0, DA_min_ = 0, DA_range_ = *θ*, RPE_min_ = −1, RPE_min_ = −1, RPE_range_ = 1; otherwise DA_min_ = *θ*, DA_range_ = 1−*θ*, RPE_min_ = 0, RPE_range_ = 1.

The baseline level of dopamine was set at a value of *θ* = 0.2 for all simulations. Initial values of the synaptic weights were set to 0.2 and action values value at 0.5, leaving the four plasticity parameters (*a*_1_, *b*_1_, *a*_2_, *b*_2_), and *α* and *β*, from the equations  and , to estimate.

The four plasticity parameters govern the magnitude of D1‐LTD (*a*_1_), D1‐LTP (*b*_1_), D2‐LTP (*a*2), and D2‐LTD (*b*_2_). Figure 2(b) gives an illustrative example of the interaction between the dopamine levels (as governed by the RPE) and its effect on striatal synaptic strength using the default plasticity coefficient parameters from Gurney et al., 2015.

#### 2.3.3 Basal ganglia model fitting—RPE parameters

At all stages of the fitting procedure, parameter optimization was performed by minimizing the negative log likelihood of the data given different values of the model's parameters. Our initial approach was to define a realistic “physiological” parameter space by fitting the control subjects’ behavior. As we wanted to eliminate any influence of the Q‐learning/RPE parameters on the eventual patient fits, we first estimated individual learning rate (*α*) and reward sensitivity (*β*) parameters for the control subjects while keeping the four plasticity parameters, (*a*_1_, *b*_1_, *a*_2_, *b*_2_) fixed at default values 0.5, 0.3, −0.5, −0.5, respectively. For each control subject, *α* and *β* estimates were derived following a grid search over the explored ranges of [0, 0.1] and [0, 0.5], respectively. The parameters derived from this grid search were then used as starting values for further optimization using Matlab's *fmincon* function. The purpose of this initial fitting procedure to the control subjects was to find values of *α* and *β* which could then be used as constants for all subsequent estimation of the plasticity coefficients. All subsequent fitting of the plasticity parameters for each subject was therefore performed while using this average value of *α* and *β* as constants. This approach was used to prevent overfitting of the behavioral data and ensure that the model fitting resulted from optimization of a four parameter search for striatal plasticity parameters, to test our hypothesis, rather than parameters relating to phasic dopamine release.

#### 2.3.4 Basal ganglia model fitting—plasticity parameters

Next we performed an exhaustive search over the entire parameter space as defined by the four plasticity parameters. The aim was to eliminate biologically unfeasible or redundant parameter space to define meaningful bounds of the constrained fitting that would follow. This approach was also found to significantly improve the consistency of parameter estimates by avoiding spurious local minima. As the “*a*” parameters are modified over a narrower range of dopamine, we allowed the search limits to be broader than the “*b*” parameters leading to ranges of [−2,2] and [−1,1], respectively. Given the four plasticity parameters could take on either positive or negative values within the parameter space, we divided this into 16 “quadrants,” each corresponding to a parameter combination with positive and negative signs. For example, the first quadrant with parameter signs [+, +, +, +] is thus defined by [0,0,0,0] for the lower bounds, [2,1,2,1] for the upper bounds. For each quadrant a coarse grid search on the parameters bounds was done followed by function minimization with *fmincon,* using the course grid search minima as starting values and the quadrant's bounds as *fmincon*'s constraints. The parameter space quadrant which produced the most reliable fits (lowest mean negative log likelihood scores) was the chosen for all subsequent analysis. This “exhaustive search” was performed for the control subjects only.

#### 2.3.5 Basal ganglia model fitting—hypothesis testing

Our aim was to establish whether pathological combinations of biologically feasible striatal plasticity abnormalities could explain differences in the patients’ behavior in the reversal learning task. Using the control estimates as normative data, parameter space bounds were defined which could be considered “pathological” and which could be used to test specific hypotheses on their ability to model patient's behavior. Specifically, we chose four separate hypothetical combinations of plasticity deficits: H_1_) increased D1‐LTP : D1‐LTD; H_2_) decreased D1‐LTP : D1‐LTP; H_3_) increased D2‐LTP : D2‐LTD; H_4_) decreased D2‐LTP : D2‐LTD. These combinations were chosen as previous in vitro experiments have demonstrated a wide range of potential cortico‐striatal plasticity abnormalities, but to date the receptor specificity of these remains unknown. With these four hypothetical combinations we could establish the most likely imbalance of cortico‐striatal plasticity in a receptor specific manner. In order to define the parameter range that corresponds to “pathological” hypothetical combinations, we fitted a nonparametric kernel to the control estimates for each of the four parameters. We then derived a probability density function from this fit to obtain the 5% and 95% confidence limits for each parameter. These were then used to determine the bounds used for the fitting of the basal ganglia model to the patients’ behavior. For example, in order to fit H_1_ (increased D1‐LTP, decreased D1‐LTD), the initial grid search was performed on a parameter space that was bounded between the 95% limit and 2 (the maximal parameter value) for “*a*_1_”; between 0 (the minimum value) and the 5% limit for “*b*_1_”, while “*a*_2_” and “*b*_2_” were constrained between the 5% and 95% confidence limit of the control parameter space. For each patient, the same procedure of grid search within the bounds defined by the hypothesis in question was followed by further optimization of the parameter estimate using *fmincon*. As the number of parameters remained fixed across each hypothesis tested (and between model structures), we compared each combination of abnormal plasticity combinations by finding the model with the minimum negative log likelihood. This fitting procedure was performed separately for the reward and loss behavioral data.

### 2.4 Statistics

Behavioral and participant demographic data were tested for normality of distribution using Kolmogorov–Smirnov test. Normally distributed data were analyzed using Student's *t*‐test with the Mann–Whitney *U* test was used as a nonparametric alternative where necessary. When more than one variable of interest was tested we used either one‐way or multivariate ANOVA. In the case of categorical data, chi‐squared test was used.

## 3 RESULTS

### 3.1 Demographics, rating scales, and behavior

The reinforcement learning task was completed by 40 patients with cervical dystonia (CD age = 55.2 ± 10.0 [range 28–72]; 28 female) and 40 sex‐ and age‐matched controls (CTRL age 54.4 ± 9.18 [range 26–74]; 27 female). The average cervical dystonia rating scale (CDIP‐58) score for the patients was 41 ± 15 (range 22–80). Patients and controls were well matched with no significant difference in average IQ as indexed by the NART (CTL 118.18 ± 3.8 CD 118.7 ± 4.1,) or Y‐BOCS scores (CTL 4.2 ± 3.1, CD 5.1 ± 4.3). There were numerically higher levels of anxiety and depression in the patients as indexed by the HADS‐A (CTL 3.5 ± 3.1, CD 5.2 ± 2.596 two‐tailed *t*‐test, t [78] = 2.4, *p* = 0.009) and HADS‐D (CTL 1.42 ± 2.3, CD 2.66 ± 2.59 two‐tailed *t*‐test, t [78] = 2.02, *p* = 0.015) but this was not clinically significant and patients did not satisfy criteria for a mood or anxiety disorder. Patients and controls completed the same number of trials of both the reward and loss avoidance task (control 238 ± 1, patients 238 ± 2.5).

**Figure 3:**
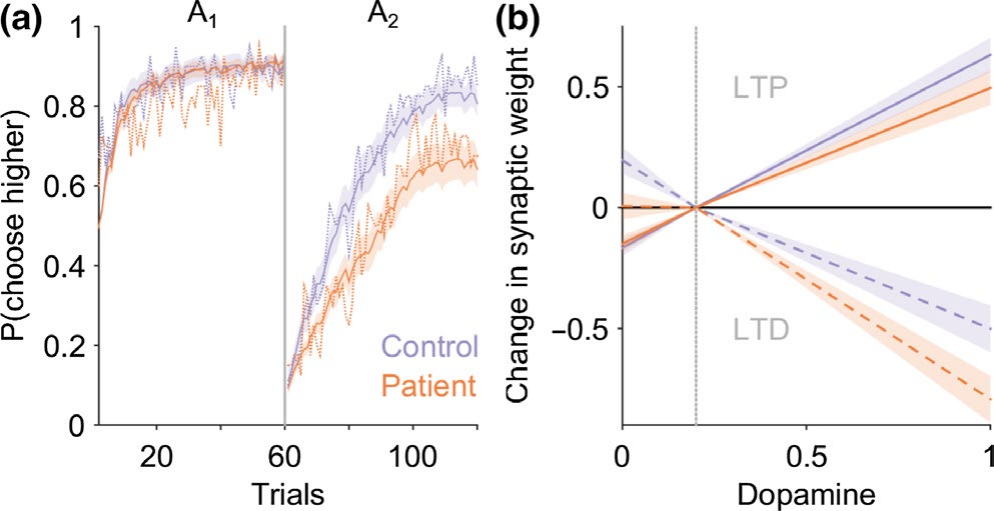
Impaired reward‐learning in cervical dystonia is explained by a model of the basal ganglia with abnormal D2 cortico‐striatal plasticity. (a) Average probability of choosing high value actions “*A*_1_” and “*A*_2_” pre‐ and post‐reversal reversal of contingencies in the reward task. Patient average choice probability in red, controls in blue. Vertical dotted line represents the point of contingency reversal. Dashed lines represent experimental behavior from patients and controls. The basal ganglia models performance following estimation of the optimal model parameters is superimposed (solid lines with 95% confidence limits represented by the shaded region). The models behavior closely overlaps with the experimental behavior of both the patients and controls. The number of rewards won post‐reversal in the reward task was significantly reduced in patients compared to controls (Mann–Whitney *z*(78) = −2.47, *p* = 0.013). A basal ganglia model with increased D2‐LTD : LTP explained the patients behavior best in 29 of the 40 patients fitted. (b) The average dopamine‐weight change curve for the patients (red) and controls (blue) with 95% confidence limits represented by the shaded region. Solid lines represent D1 and dashed lines represent D2 curves, respectively. During phasic “bursts” in dopamine the patients undergo significantly greater D2‐LTD and correspondingly reduced D2‐LTP during phasic “dips” below baseline (vertical gray dashed line) [Colour figure can be viewed at http://wileyonlinelibrary.com]

**Figure 4:**
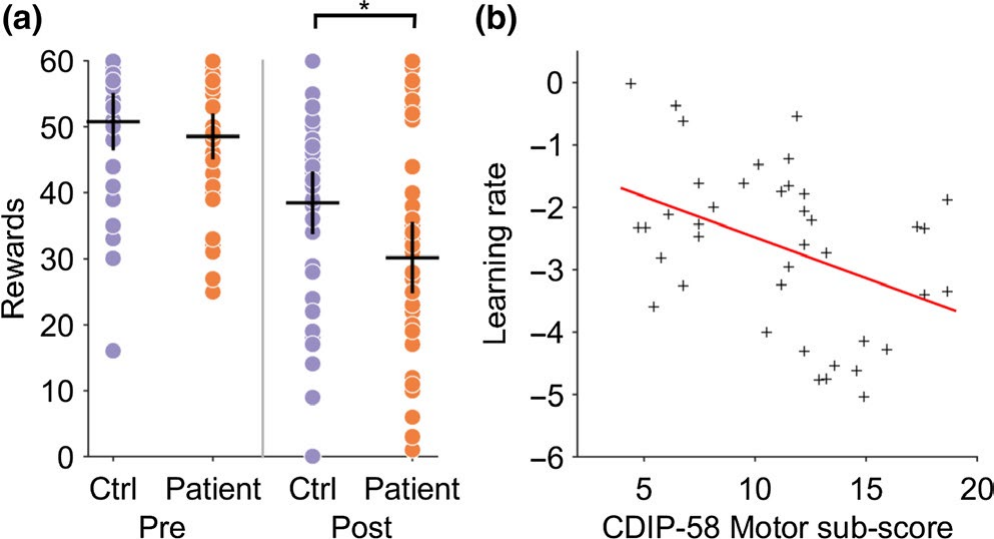
Cervical dystonia reward‐learning is impaired and correlates with severity of motor symptoms. (a) Individual performance in patients (red dots) and controls (blue) demonstrates significant reduction in number of rewards obtained post‐reversal (*p = 0.013, Mann–Whitney *z*(78) = −2.47). Black crosses represent mean and 95% confidence limits. (b) The normalized learning rate (*σ* ^‐1^ *α*) estimate for each individual patient is plotted against their CDIP‐58 motor sub‐scores. There was a significant negative relationship between motor score severity and learning rate (*R* ^2^ = 0.17, *p* = 0.008) [Colour figure can be viewed at http://wileyonlinelibrary.com]

**Figure 5:**
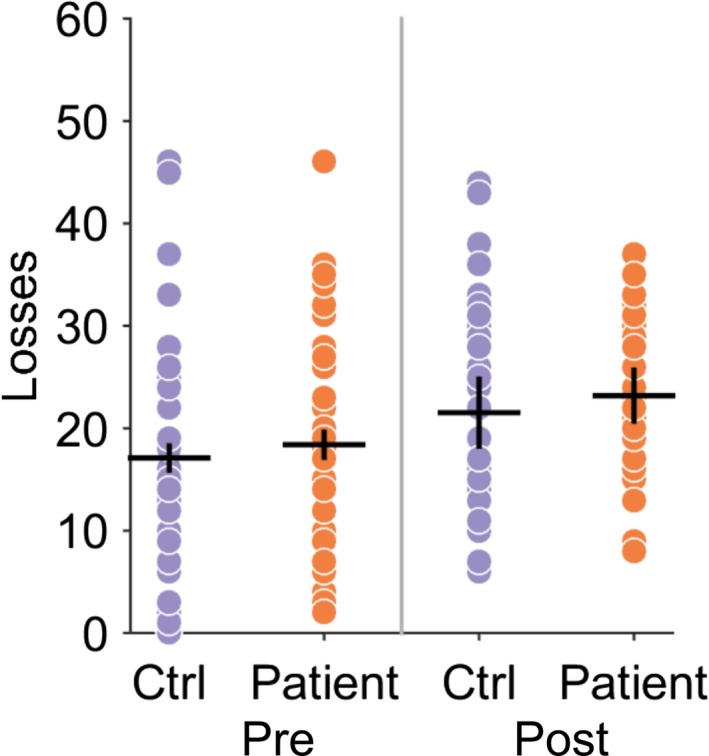
Loss avoidance learning in patients and controls. Individual performance in patients (red dots) and controls (blue) demonstrates no significance difference in loss avoidance performance. Black crosses represent mean and 95% confidence limits [Colour figure can be viewed at http://wileyonlinelibrary.com]

Patients had a selective post‐reversal deficit in reward‐learning (Figure 3a). A one‐way ANOVA demonstrated a significant between‐group difference in the performance of the reward task (*F*[1,78] = 4.62.9, *p* = 0.03). The number of rewards won pre‐reversal was similar between patients and controls, (CD = 50 ± 4.0, CTL 48 ± 3.2, Mann–Whitney *z* (78) = −1.61, *p* = 0.11); however, the number of rewards won post‐reversal were significantly less for patients (CD = 30.1 ± 5.1, CTL = 38 ± 4.5, Mann–Whitney *z* (78) = −2.47, *p* = 0.013) implying an impairment in reward‐based reversal learning (Figure 4a). No difference in the pre‐ or post‐reversal learning performance in the loss avoidance task between patients and controls was observed (one‐way ANOVA between‐group difference *F*(1,68) = 1.15, *p* = 0.28), suggesting that learning from positive (reward) rather than negative (aversive) feedback was selectively impaired in patients with cervical dystonia (Figure 5).

### 3.2 Reinforcement learning model fitting

To test the hypothesis that phasic dopamine signaling was abnormal in patients we modeled subject choice data from the reward behavioral task using a trial‐by‐trial reinforcement learning model. This model was fitted to each individual participant's behavioral choices in the reward task. A significant reduction in the learning rate parameter (α) was observed in the patients (CD α = −2.63 ± 1.29, CTL, α = −1.92 ± 0.99, two‐tailed *t*‐test *p* = 0.007, *t*[78] = −2.7) but not in the reward sensitivity parameter *β* (CD, *β* = 1.7 ± 0.5, CTL, 1.8 ± 0.56, *t*[78] = −0.7, *p* = 0.42). This is consistent with the patients placing less weight on the prediction error signal, as the learning rate parameter α multiplies the prediction error term in the trial‐by‐trial reinforcement learning model. No correlation was observed between individual values of α and the total CDIP‐58 score (*R* ^2^ = 0.07, *p *= 0.132). However, a negative correlation between the “Head and Neck” symptom sub‐score was present (*R* ^2^ = 0.17, *p* = 0.008), meaning patients with the most severe motor symptoms tended to have the lowest learning rates (Figure 4b), and hence were most impaired at learning from positive feedback. We found no correlations with the learning rate parameter and the other CDIP‐58 sub‐scores, which include global estimates of disease impact on quality of life and psychosocial wellbeing, supporting a specific relationship between impaired reversal learning and motor symptoms in these patients. No correlation was found between the reward sensitivity parameter *β* and total CDIP‐58 clinical rating scale or its sub‐scores.

### 3.3 Event‐related fMRII analysis

**Figure 6:**
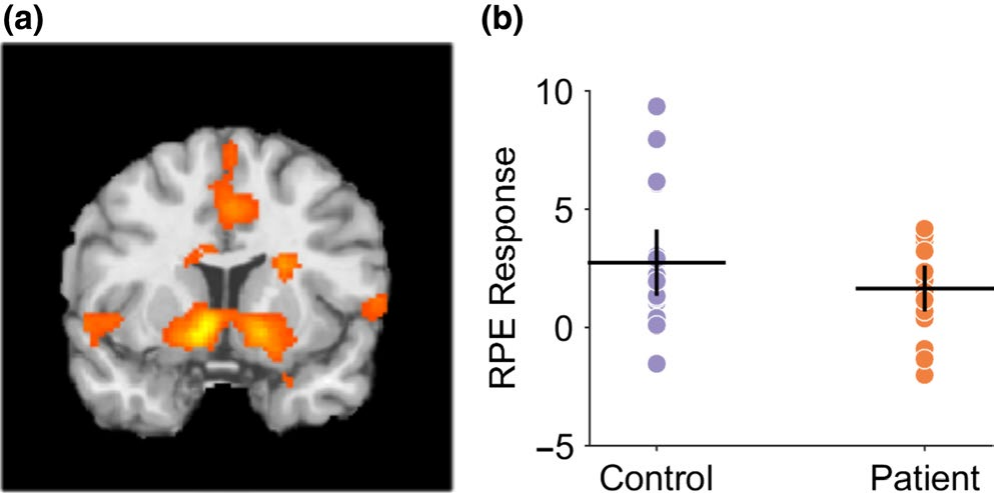
Reward prediction error encoding in controls and patients during reversal learning task. (a) Controls exhibited prediction error encoding within the striatum with maximal clusters in the left putamen, MNI co‐ordinates [−10,10,−6], *T*‐value = 5.14, and right caudate nucleus, MNI co‐ordinates [10,7,−10], *T*‐value = 5.08, both *p* < 0.01, cluster extent corrected across the whole brain. There was no significant between‐group differences (at *p* = 0.05) suggesting comparable prediction error encoding in patients and controls. (b) Region of interest analysis from left putaminal RPE‐fMRI signal in controls and patients with mean and 95% confidence limits supporting comparable levels of RPE signaling in both groups [Colour figure can be viewed at http://wileyonlinelibrary.com]

Blunted RPE signaling (as indexed by a lower learning rate) might be an explanation for the impairment in reward‐based learning in these patients and has been proposed for different patient groups. However, the results of an event‐related model‐based fMRI analysis of participants demonstrate that RPE signaling within the basal ganglia was intact in patients and at levels comparable to controls (Figure 6). Controls exhibited prediction error encoding within the striatum with maximal clusters in the left putamen, MNI co‐ordinates [−10,10,−6], *T*‐value = 5.14 and right caudate nucleus (MNI co‐ordinates [10,7,−10], *T*‐value = 5.08, both *p* < 0.01, cluster extent corrected across the whole brain. There was no significant between‐group differences (at *p* = 0.05) suggesting comparable RPE encoding in patients and controls.

### 3.4 Basal ganglia model fitting

#### 3.4.1 Controls

Our fMRI result suggests that (presynaptic) phasic dopamine signaling is normal in patients and a more likely explanation for impaired reversal learning is abnormal postsynaptic modification in cortico‐striatal strength. To further understand this potential mechanism, the “Basal Ganglia” model was used which, as above, incorporated standard reinforcement learning rules with details of cortico‐striatal plasticity (Figure 2), allowing inference of synaptic weight changes pertaining to direct (D1) and indirect (D2) pathway potentiation (LTP) and depression (LTD). Six parameters were estimated; two relating to a standard reinforcement learning model (the learning rate [α] and reward sensitivity [*β*] parameters) and four plasticity coefficients representing D1‐LTD, D1‐LTP, D2‐LTP, and D2‐LTD represented by (*a*_1_, *b*_1_, *a*_2_, *b*_2_), respectively (Figure 2b). All initial fitting was performed on the control behavior in order to define the “physiological” parameter space prior to testing hypothetical pathological explanations for the patients abnormal behavior. As the purpose of this model was to test for abnormal postsynaptic plasticity abnormalities, here we fixed the learning rate (*α*) and reward sensitivity (*β*) parameters, which govern the phasic dopamine signals dynamics, to constant values. The final estimated α and *β* values estimated for the 40 control subjects were α = 0.036 ± 0.05, *β* = 0.28 ± 0.14 for reward‐learning, and α = 0.11 ± 0.22, *β* = 0.36 ± 0.24 for loss avoidance. All subsequent fitting of the model used these average values while allowing the remaining four plasticity parameters to vary. By excluding the patients from this analysis, this further reinforced the a priori assumption of our model fitting and results of the fMRI analysis, that phasic RPE signaling was intact and physiologically comparable to control levels, ensuring that any difference in behavior was explained by differences in plasticity parameters.

**Figure 7:**
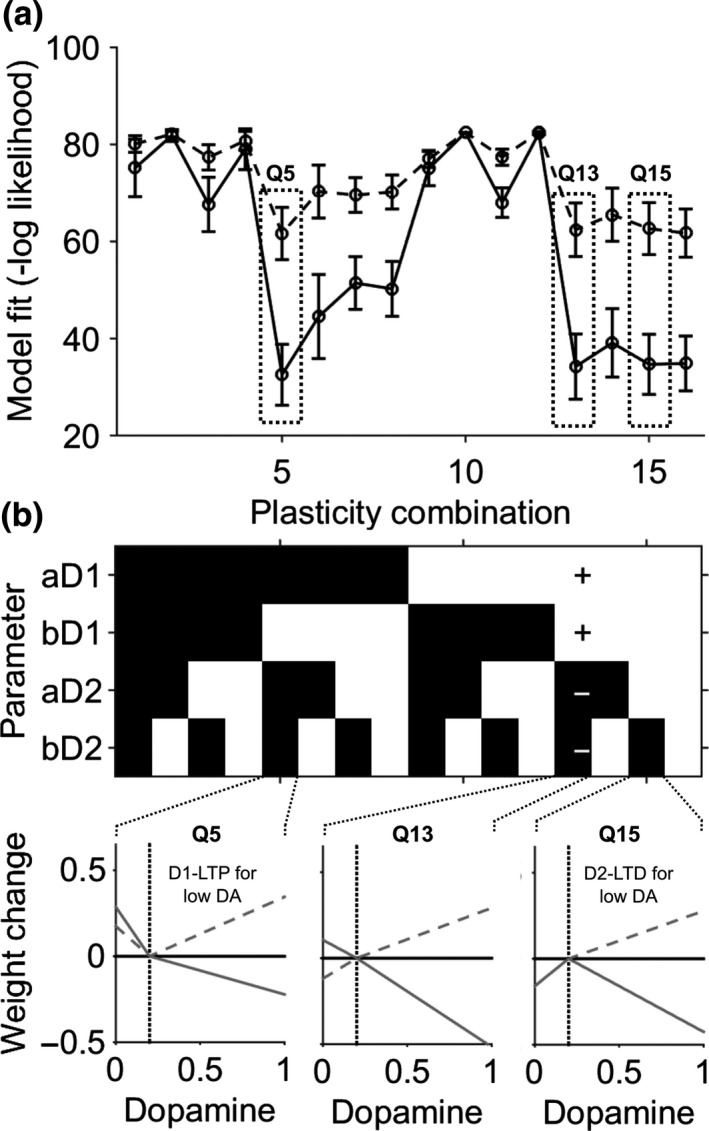
Quadrant parameter space mapping. (a) The average negative log likelihood (error bars represent 95% confidence intervals) for model fitting to the control behavior. Solid lines represents reward fitting, dashed lines results from fitting the loss avoidance data. By constraining the plasticity parameters to one of 16 “quadrants,” consistent regions of parameter space redundancy were identified. (b) Illustrative examples of dopamine‐weight change curves derived from quadrants which generated good fits (Q5 and Q15) to the control behavior but biologically unrealistic changes (left panel D1 LTP occurred during a dopamine “dip”, right panel D2‐LTD occurred during a dopamine “dip”). Solid lines represent the D2 curve, dashed lines the D1 curve. Quadrant 13, where the parameter space was bound by signs [+1 +1 −1−1] generated the most reliable fits with biologically feasible weight change curves (middle panel example)

**Figure 8:**
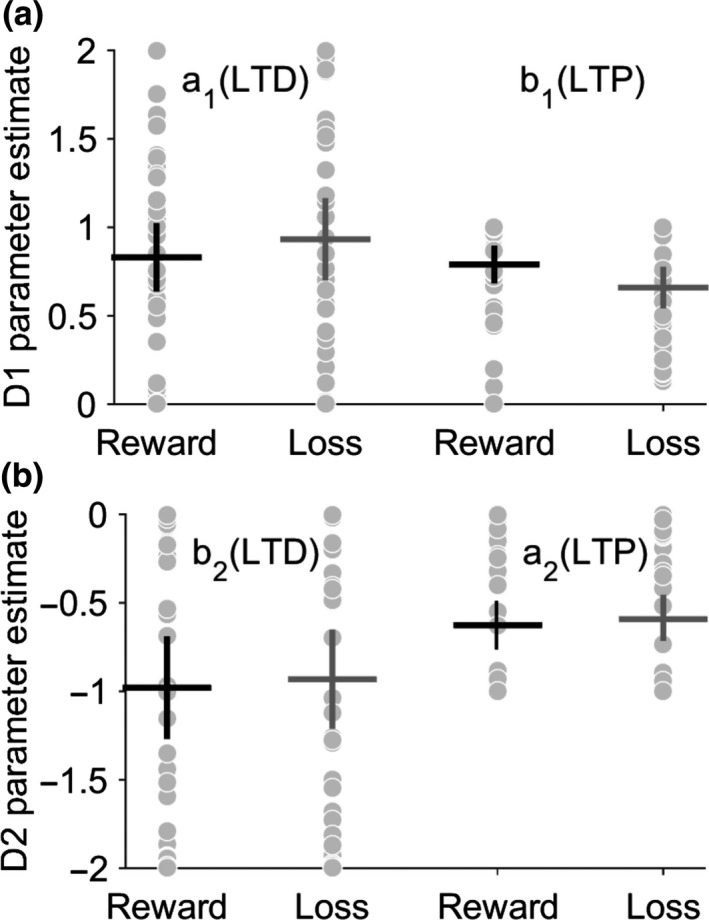
Basal ganglia model fitting recovers realistic plasticity parameter estimates for controls. Final plasticity parameter estimates for D1 in (a) and D2 (b) coefficients. Reward and loss avoidance were fitted separately with individuals estimates represented by gray dots. Mean and 95% confidence limits represented by black and gray crosses for reward and loss, respectively. The average plasticity parameter values were *a*_1_ = 0.83 ± 0.55, *b*_1_ = 0.78 ± 0.27, *a*_2_ = −0.97 ± 0.85, *b*_2_ = −0.62 ± 0.37 for reward and *a*_1_ = 0.93 ± 0.67, *b*_1_ = 0.65 ± 0.31, *a*_2_ = −0.93 ± 0.82, *b*_2 _= −0.59 ± 0.38 for the loss avoidance task

Next an exhaustive search over the entire parameter space was done defined by systematic variation of the four plasticity parameters eliminating biologically unfeasible or redundant parameter space in order to define the bounds of the constrained fitting that would follow. This analysis (Figure 7) demonstrated that only a small number of parameter combinations modeled the behavior well, the majority fitting the behavior poorly. Of the few parameter combinations which did model the behavior consistently well, only one was consistent with the known physiological plasticity modifications of dopamine at D1 and D2 synapses. We therefore settled on constraining the parameter bounds to within this territory of the parameter space where *a*_1_ = [0,2],*b*_1_ = [0,1],*a*_2_ = [−2,0],b_2_ = [−1,0]. The final plasticity parameter estimates for the controls (Figure 8) accurately reproduced controls subject's behavior (Figure 3a), and dopamine‐synaptic weight change curves were generated (Figure 3b), comparable to those observed by Gurney et al. (2015) (their fig. 13).

#### 3.4.2 Patients

In order to test whether abnormal D1 (direct) or D2 (indirect) pathway cortico‐striatal plasticity could explain the reward‐learning impairment in patients, four candidate hypothetical abnormalities of synaptic plasticity were tested: (H_1_) increased D1‐LTP : D1‐LTD; (H_2_) decreased D1‐LTP : D1‐LTP; (H_3_) increased D2‐LTP : D2‐LTD; (H_4_) decreased D2‐LTP : D2‐LTD. Each of these hypotheses was tested in turn by fitting the basal ganglia model to the patient's behavioral data.

Comparing the negative log likelihood estimate for each hypothesis, H4 (decreased D2‐LTP : D2‐LTD) fitted the patient's behavior best in 29 out of 40 patients for the reward task, with the next best hypothesis H1 (increased D1‐LTP : D1‐LTD) in 11 of 40, chi‐square (1) = 14.45, *p* < 0.001. Notably in the loss avoidance task, despite no significant difference in behavioral performance, H4 (decreased D2‐LTP: D2‐LTD) also best explained patient's behavior in 34 of 40 subjects, with the remaining six subjects best explained by H1 (increased D1‐LTP : D1‐LTD), chi‐square (1) = 36.45, *p* < 0.001. Thus, a single deficit of decreased D2‐LTP: D2‐LTD is consistent with both the patients’ worse reversal performance in the reward task and their reversal performance in the loss avoidance task.

**Figure 9:**
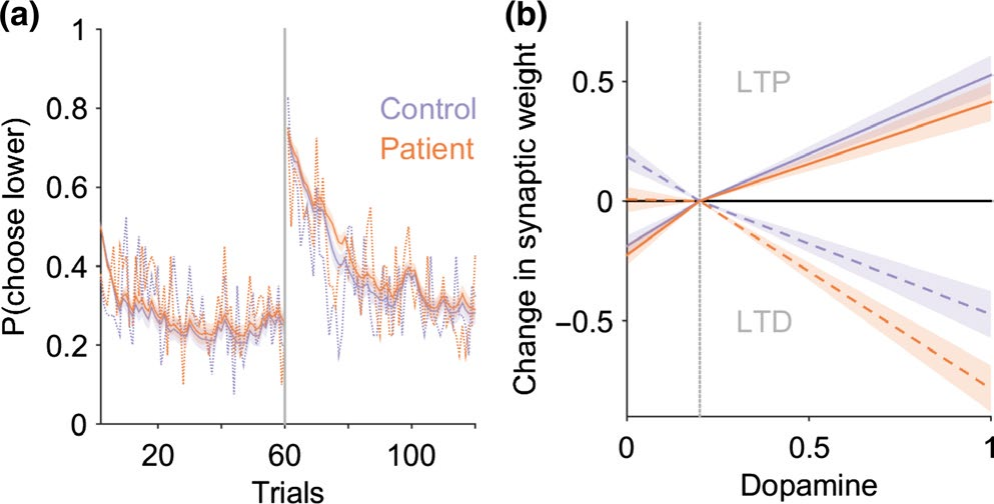
Loss avoidance learning in cervical dystonia is best explained by a model of the basal ganglia with abnormal D2 cortico‐striatal plasticity. (a) Average probability of choosing low value actions “A_1_” and “A_2_” pre‐ and post‐reversal reversal of contingencies in the reward task. Patient average choice probability in red, controls in blue. Vertical dotted line represents the point of contingency reversal. Dashed lines represent experimental behavior from patients and controls. The basal ganglia models performance following estimation of the optimal model parameters is superimposed (solid lines with 95% confidence limits represented by the shaded region). The models behavior closely overlaps with the experimental behavior of both the patients and controls. Despite no behavioral difference in the loss avoidance learning, a basal ganglia model with increased D2‐LTD : LTP explained the patients loss avoidance strategy best in 31 of the 40 patients fitted. (b) The average dopamine‐weight change curve for the patients (red) and controls (blue) with 95% confidence limits represented by the shaded region. Solid lines represent D1 and dashed lines represent D2 curves, respectively [Colour figure can be viewed at http://wileyonlinelibrary.com]

**Figure 10:**
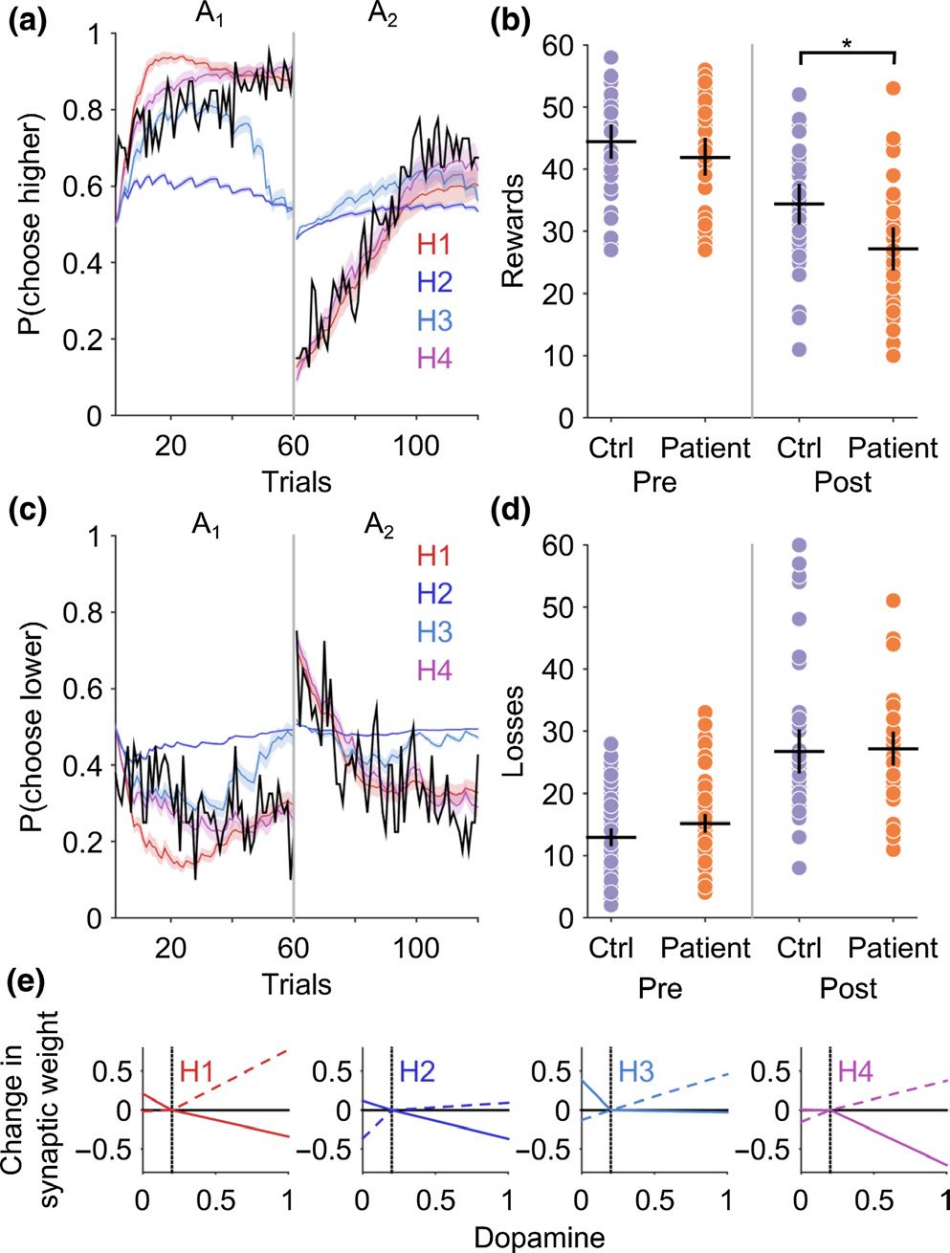
Model hypotheses comparison and generated behavior in reward and loss avoidance tasks. (a) and (c) The mean choice probability for the high and low value fractal for the patients is plotted in black. Superimposed are the result of fitting the basal ganglia model to each of the four hypothetical plasticity abnormalities (H1, D1 increased LTP : LTD; H2, D1 increased LTD : LTP; H3, increased D2 LTP : LTD; H4, increased D2 LTD : LTP). Average model choice probabilities with 95% confidence limits represented by the shaded area. (b) Behavioral data generated by the basal ganglia model performing the task with the “winning” hypothesis H4 (increased D2 LTD: LTP) reproduces the post‐reversal reward impairment (*p < 0.01) with similar loss avoidance performance in patients and controls (d). (e) Final dopamine‐weight change curves for each of the four hypothetical abnormalities in D1 or D2 cortico‐striatal plasticity. Dashed lines D1, solid lines D2 [Colour figure can be viewed at http://wileyonlinelibrary.com]

Overlaying observed experimental choice behavior with the final best model fit demonstrated a close correspondence to both the reward (Figure 3a) and loss avoidance (Figure 9a), in both controls and patients. In contrast, there was a poor overlap between the modeled patients choice in both tasks when alternative plasticity hypotheses were fitted (H1–H3)—see Figure 10 (a & c).

Using the final optimal parameter estimates for patients (using hypothesis H4) and controls, simulated behavioral data were generated using the basal ganglia model performing the original task. This simulated behavior reproduced both the post‐reversal impairment in the patients for the reward task (Figure 10b) and performance in the loss avoidance task (Figure 10d).

Overall, this analysis and results provides strong evidence in support of a pathological abnormality of D2 cortico‐striatal plasticity, with excess synaptic depotentiation leading to a measurable impairment in reward reversal learning.

## 4 DISCUSSION

This is the first study to demonstrate that patients with cervical dystonia have impaired reinforcement learning. A novel reinforcement learning basal ganglia model was developed which, when fitted to control subjects’ behavior, successfully recovered measurable and biologically realistic synaptic parameters. Our results suggest that abnormal reversal learning in patients with cervical dystonia is best explained by abnormally high levels of D2 synaptic depotentiation. Notably, striatal D2 receptors are thought to be critical to reversal learning, as they are sensitive to the pause in dopamine release that accompanies trials following the reversal where reward is expected but not forthcoming (Cools et al., 2009; Cox et al., 2015; Frank, 2005). Selective blockade of D2 receptors in humans, or destruction of D2 expressing MSN's in animals, leads to impairments in the reversal learning phase of reward‐learning tasks (Mehta, Swainson, Ogilvie, Sahakian, & Robbins, 2001; Nakanishi et al., 2014). Our reversal learning impairment in this group of patients is therefore consistent with these observations. Abnormal D2‐receptor function is also consistent with SPECT studies reporting a selective reduction in striatal D2 receptor expression (Naumann et al., 1998) and the recognized clinical relationship between chronic D2 receptor blockade and tardive dystonia (Sethi & Morgan, 2007). Furthermore, D2 receptor dysfunction has also been demonstrated in other forms of focal dystonia including spasmodic dysphonia and writer's cramp (Berman, Hallett, Herscovitch, & Simonyan, 2013; Simonyan, Berman, Herscovitch, & Hallett, 2013), suggesting that abnormal reward‐learning mediated via impaired D2 receptor function would be relevant to a broad range of focal dystonia phenotypes. More recently, increased lysosomal degradation has been identified as the molecular basis for decreased D2 receptor density in DYT1 dystonia, once again reinforcing the increasing central convergent role for D2R dysfunction in dystonia (Bonsi et al., 2019). The extent to which these results can be extended to focal forms, such as cervical dystonia, remains unclear; however, it is interesting to note that recently identified genes associated with a cranio‐cervical phenotype, including GNAL, THAP1, and ANO3, share common roles in striatal signal transduction (Charlesworth et al., 2012; Kumar et al., 2014; Zakirova et al., 2018).

Our modeling supports the idea that the reversal learning impairment in these patients could be caused by a relative absence of D2‐LTP associated with the “dip” in dopamine particularly at the time of contingency reversal. The insufficient “NoGo” activity that would be a consequence of this would result in a delay to suppress the decision to choose the opposite action with high pre‐reversal value. Furthermore, our results predict that such NoGo activity required to suppress a previously learnt choice has to rise from an initially depotentiated state. This is a direct consequence of excessive D2‐LTD that accompanies the “bursts” of dopamine associated with the pre‐reversal acquisition phase of the task. This mechanism therefore suggests perseveration errors that led to poorer task performance were mediated by impaired response inhibition, rather than over learning (increased LTP) or an inability to extinguish the previously learnt action values (impaired LTD). This distinction is important, as all of the four hypothesis tested could have provided equally plausible mechanistic explanations for the observed abnormal behavior. This interpretation is also compatible with the recent suggestion (Sadnicka et al., 2017) that impairments in a temporal discrimination task in cervical dystonia could represent an increase in decision boundary with patients requiring more evidence before making a decision. Conceivably, if the negative prediction error signal which highlights a novel unexpected change (such as a change in the interstimulus interval) cannot induce sufficient D2‐LTP, additional sensory evidence and repeated presentation of the same deviant stimulus will be required to decide that the interval has changed. This is potentially analogous to the additional perseverative choice trials post‐reversal that our patients performed before making the decision to “reverse” their choice of action.

How do these results relate to previous examples of abnormal LTP and LTD‐like plasticity in focal forms of dystonia? Although direct comparison between noninvasive brain stimulation paradigms and the inference of abnormal cortico‐striatal plasticity from reinforcement learning is difficult, our modeling provides support to the more general idea that impaired synaptic plasticity may be a trait marker of focal forms of dystonia. Furthermore, given that increased cortical LTP‐like plasticity has been the most consistent finding in these studies (Quartarone & Pisani, 2011), it would seem likely that potentially complex combinations of cortical and subcortical derangements in synaptic plasticity contribute to the pathophysiology of focal dystonia syndromes.

Our results extend previous work (Arkadir et al., 2016) which demonstrated abnormal reward‐learning in patients with DYT1 generalized dystonia. In addition, they support a more general conclusion that abnormal reward‐based learning may be a common phenotypical abnormality in both generalized and common focal forms of dystonia. This parallels the original clinical observations of Marsden and Harrison (Marsden & Harrison, 1974), who realized the overlapping features between “spasmodic torticollis” and “idiopathic Torsion dystonia”. Although we propose a shared substrate of abnormal striatal plasticity may contribute to the similarities clinically between generalized and focal forms of dystonia, it is unlikely that these abnormalities of striatal function are sufficient in isolation to cause dystonic action selection degradation. An important future task of further research therefore will be reconciling the proposed striatal plasticity abnormalities here with recent models of cervical dystonia, such as the recently proposed neural integrator theory (Shaikh, Zee, Crawford, & Jinnah, 2016). One potential point of convergence with this work is the top‐down influence of biased striatal plasticity on the pallidal output neurons as these have been proposed to modulate the brainstem neural integrator which controls head position. A future avenue that may prove useful for exploration, potentially via simulation, is whether asymmetries in GPi output can arise from upstream striatal plasticity abnormalities, as asymmetric GPi firing rates have been shown to correlate with the severity and direction of torticollis (Moll et al., 2014; Sedov et al., 2019).

There are some possible study limitations. Computational modeling cannot be used to make definitive statements about synaptic level phenomena which is only directly accessible to in vitro neurophysiological techniques. Its crucial role is to generate hypotheses about disease mechanisms, which can be tested by experimental replication and manipulation of behavior based on the model's predictions, and by interpretation of clinical trials of novel medications with known effects on synaptic plasticity. In the absence of a clearly defined genetic cause and corresponding animal model for the commonest forms of focal dystonia, it is unlikely that any other approach can be used to address the hypotheses we had for this study due to ethical considerations. As described previously in patients with cervical dystonia, we found higher levels of anxiety and depressive thoughts. These were at levels which were did not reach the threshold for being clinically significant for mild depression or anxiety and are unlikely to be responsible for the behavioral impairment observed as impaired reversal learning has only been observed in patients with severe depression or bipolar disorder (Dickstein et al., 2010).

The study results have potential therapeutic implications for developing new treatments for patients with cervical dystonia. As D2‐LTP is promoted by low dopamine states drugs which deplete dopamine (such as tetra‐or valbenazine) would be expected to improve reversal learning in these patients and may ultimately have a therapeutic role in the treatment of dystonia. For the third of patients who receive no benefit from existing treatments (Misra, Ehler, Zakine, Maisonobe, & Simonetta‐Moreau, 2012), future studies which combine reward‐learning tasks with a single‐dose pharmacological challenge may be used to identify new agents, based on their ability to improve reward learning behavior. Furthermore, this approach could be used to stratify subgroups of patients according to their response, so that personalized treatments with disease modifying potential can be selected for future clinical trials.

## 5 CONCLUSIONS

In summary, we report a behavioral reversal learning deficit in patients with cervical dystonia, extending previous work on DYT1 dystonia. Abnormal reversal learning behavior was best explained by decreased D2‐LTP, suggesting excessive D2 cortico‐striatal synaptic depotentiation. Computational modeling of behavior in patients can be used to test hypotheses of abnormal synaptic plasticity inaccessible by other means and thus to interpret the behavioral effects of novel treatments.

## COMPETING INTERESTS

None of authors have any competing interests, financial, or otherwise to declare.

## AUTHOR CONTRIBUTIONS

TG and DS designed research. TG and MH contributed to analytical tools and performed analysis of the data. TG, DS, and MH wrote the paper.

## Data Availability

The code and behavioral data can be requested by email to the corresponding author (TG).
