# Supplementary material for: Maladaptive striatal plasticity and abnormal reward‐learning in cervical dystonia
Source: Eur J Neurosci. 2019 May 14;50(7):3191–204. doi: 10.1111/ejn.14414 (PMC6900037; doi:10.1111/ejn.14414)
Supplement: Supplementary file 1 [file EJN-50-3191-s001.pdf]

Supporting Information

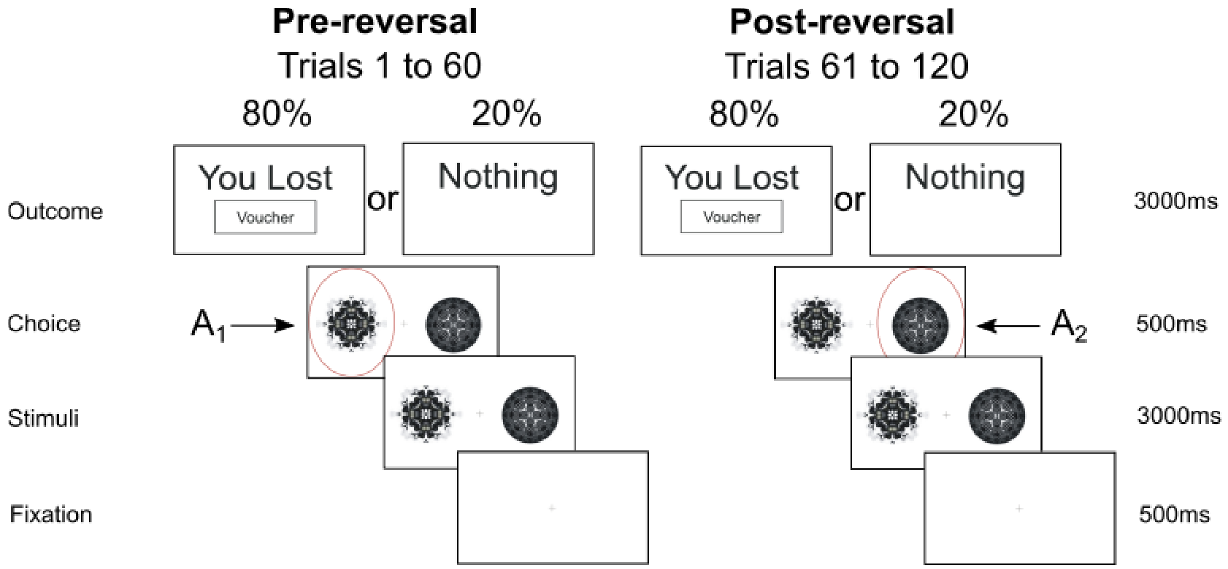

Supplementary Figure 1:- Probabilistic reversal learning task. Example of fractal images presented during a single loss trial. The probability of receiving a loss "voucher" reverses after 60 trials requiring the participant to suppress the previously learnt choice and learn to "reverse" their decision to choose the previously low value (pre-reversal) fractal. In the pre-reversal phase choice of A_1 is associated with an 80% outcome of loss. Post- reversal this reduces to 20% with the choice of A_2 being associated with 80%.
